# Single-Cell Transcriptome and Pigment Biochemistry Analysis Reveals the Potential for the High Nutritional and Medicinal Value of Purple Sea Cucumbers

**DOI:** 10.3390/ijms241512213

**Published:** 2023-07-30

**Authors:** Lili Xing, Lingyu Wang, Shilin Liu, Lina Sun, Gary M. Wessel, Hongsheng Yang

**Affiliations:** 1 CAS Key Laboratory of Marine Ecology and Environmental Sciences, Institute of Oceanology, Chinese Academy of Sciences, Qingdao 266071, China; lilixing@qdio.ac.cn (L.X.); shlliu@qdio.ac.cn (S.L.); hshyang@qdio.ac.cn (H.Y.); 2 CAS Engineering Laboratory for Marine Ranching, Institute of Oceanology, Chinese Academy of Sciences, Qingdao 266071, China; 3 University of Chinese Academy of Sciences, Beijing 100049, China; 4 Department of Biology, Duke University, Durham, NC 27708, USA; hswanglingyu@cspc.cn; 5 Department of Molecular Biology, Cellular Biology, and Biochemistry, Brown University, Providence, RI 02912, USA

**Keywords:** single-cell RNA-seq, pigmentation, sea cucumber, melanin, quinone pigments, nutritional and medicinal value

## Abstract

The sea cucumber *Apostichopus japonicus* has important nutritional and medicinal value. Unfortunately, we know little of the source of active chemicals in this animal, but the plentiful pigments of these animals are thought to function in intriguing ways for translation into clinical and food chemistry usage. Here, we found key cell groups with the gene activity predicted for the color morphology of sea cucumber body using single-cell RNA-seq. We refer to these cell populations as melanocytes and quinocytes, which are responsible for the synthesis of melanin and quinone pigments, respectively. We integrated analysis of pigment biochemistry with the transcript profiles to illuminate the molecular mechanisms regulating distinct pigment formation in echinoderms. In concert with the correlated pigment analysis from each color morph, this study expands our understanding of medically important pigment production, as well as the genetic mechanisms for color morphs, and provides deep datasets for exploring advancements in the fields of bioactives and nutraceuticals.

## 1. Introduction

Animal pigmentation and the resulting body colors influence numerous biological functions related to survival and reproduction [1], including sexual selection, concealment, mimicry, warning of toxicity, thermoregulation, photoprotection, and linkage to beneficial characteristics such as immunity, anti-drying and salinity tolerance [2]. Studying pigmentation has expanded our understanding of evolution, genetics, and developmental biology [3]. It can inform the generation and maintenance of phenotypic diversity, and the pigmentation system is an ideal phenotype to explore connections between genotype and phenotype for ecologically important traits. Body color variation has served as a model for understanding local adaptation and ecologically mediated divergence and speciation [3]. However, research on pigmentation and body color in echinoderms is scarce, and research on the origins, differentiation, and differences between species or varieties of pigment cells is lacking.

Sea cucumbers not only have important nutritional and medicinal value, but also perform important ecological functions in the energy cycle of marine substances [4]. *Apostichopus japonicus*, the most economically important species of sea cucumber, is consumed widely in China, and it has a long dietary and cultural history. Acquisition of pigmentation is an important stage in the growth and development of *A. japonicus*, and body color is one of the most important traits in selective breeding because it can affect taste and market price [5,6]. In China, sea cucumbers are generally green, and the more desirable purple sea cucumbers are rare in nature and have high potential market value. Research on pigmentation could support the breeding of specific varieties of sea cucumber, as well as seedling production.

Sea cucumbers comprise the holothuroidea taxon in the phylum Echinodermata, and their body color diversity provides an ideal trait for studying pigmentation and environmental adaptation. Echinoderms belong to deuterostomes, closely related to chordates; hence, many genes involved in pigmentation in echinoderms are more closely related to homologs in mammals than those in other invertebrates. Research on the regulatory mechanisms of pigmentation in echinoderms can link studies on invertebrate and vertebrate body color, essential for an understanding of pigmentation, body color polymorphism, and evolutionary biology.

Transcriptome analysis enables mapping of genotypes to phenotypes. However, typical transcriptome analysis experiments are likely to miss important cell-to-cell variability due to the assumption that cells from a given tissue are homogeneous. Single-cell RNA sequencing (scRNA-seq) combines advances in microfluidics and nucleic acid biochemistry to identify genes expressed within single cells [7]. ScRNA-seq can reveal complex and rare cell populations, uncover regulatory relationships between genes, and track the trajectories of distinct cell lineages in development [8]. Perillo et al. (2020) used scRNA-seq to reveal two distinct groups of pigment cells in the purple sea urchin *Strongylocentrotus purpuratus* and tested the role of the transcription factor glial cells missing (gcm) in pigment cell development. This work provided large datasets for exploring evolutionary selection of the biosynthesis of pigmentation [9]. Paganos et al. (2021) used scRNA-seq to survey cell types in early pluteus larvae in *S. purpuratus*, and they identified 21 distinct cell clusters, representing cells of digestive, skeletal, immune, and nervous systems [10]. Massri et al. (2021) used scRNA-seq to study transcriptional changes in cell states in the embryos of the sea urchin *Lytechinus variegatus* during development to the larval stage [11]. These approaches in the Echinoidea taxon of Echinodermata provide excellent leverage for the comprehensive reconstruction of transcriptional trajectories in the closely related sea cucumber during its pigmentation processes.

In the present study, we used scRNA-seq to dissect cellular heterogeneity and transcriptional dynamics in pigmentation in green and purple body wall color morphs of the sea cucumber *A. japonicus*, and we identified 26 cell clusters. We inferred gene signatures and specific marker genes for the main cell types in this tissue, and profiling individual cells at two different developmental stages enabled us to reconstruct developmental trajectories of pigmentation. In addition, combining cell type-specific and color morph-specific genes revealed by the scRNA-seq dataset allowed us to uncover new regulators of body color polymorphism with high accuracy. We then integrated results of the analysis of pigment biochemistry with the transcript profiles to illuminate the molecular mechanisms regulating distinct pigment formation in echinoderms.

## 2. Results

### 2.1. Transcriptional Clustering of Cell Populations in the Body Wall of the Sea Cucumber

**Figure 1:**
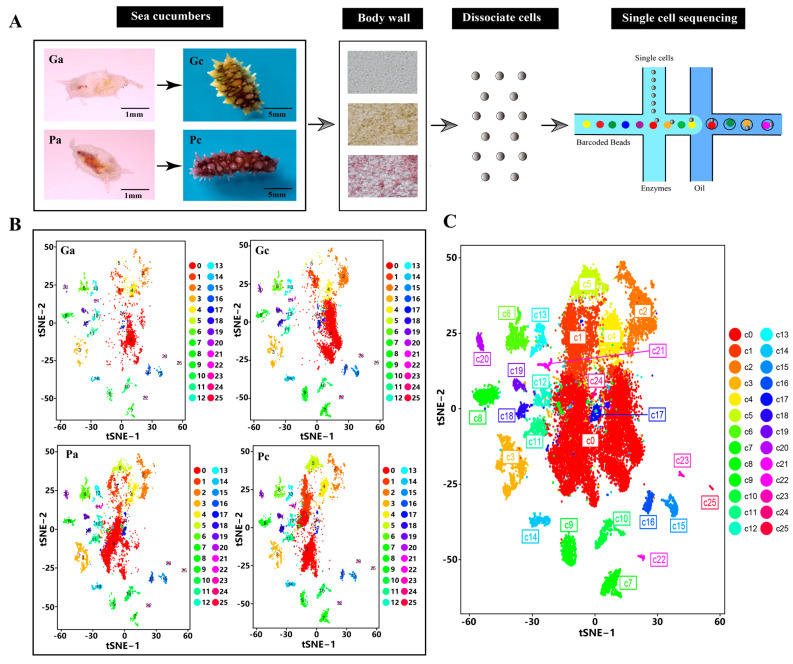
Sea cucumber body-wall single-cell sequencing identified 28 distinct clusters. (**A**) Schematic diagram of the experimental design. (**B**) Visualization of the 27,346 sequenced cells from Ga, Gc, Pa, and Pc in t-SNE plot. (**C**) Cells of all four samples are color coded by cluster.

We isolated body wall samples from four groups of animals: green and purple sea cucumbers in the early pigmentation stage (Ga, Pa), and green and purple sea cucumbers in the late pigmentation stage (Gc, Pc). Each sample comprised body wall tissue from three sea cucumbers. All samples were then prepared for scRNA-seq using the 10× Chromium platform (Figure 1A). Sample sequencing quality is shown in Table S1, and read statistics for each sample are listed in Table S2. After quality filtering, we detected a median of 600 genes and 1079 transcripts (UMI counts) from 7915 cells in Pa, a median of 562 genes and 1001 transcripts from 8839 cells in Pc, a median of 733 genes and 1286 transcripts from 6871 cells in Ga, and a median of 745 genes and 1367 transcripts from 7818 cells in Gc (Table S3). Cells were visualized using global t-distributed stochastic neighbor embedding (t-SNE) plots. We found an abnormal cell cluster in the Ga sample that we thought might be either cell aggregates or non-body-wall cells, and this cluster was not analyzed further. Body-wall cells were clustered based on transcriptional similarity, resulting in 26 transcriptionally distinct cell clusters covering green and purple sea cucumbers at two different pigmentation stages (Figure 1B,C).

Among the different cell clusters, cluster 0 contained the greatest number of cells (Table S4), representing 40.31%, 31.25%, 45.38%, and 44.48% of total cells sequenced in Pa, Pc, Ga, and Gc, respectively. More than 65% of cells were contained in the largest five clusters, whereas seven of 26 clusters represented <1% of the total sequenced cells (Table S4). For purple sea cucumbers in the early pigmentation stage, clusters 0, 1, 2, 3, and 6 were the largest, accounting for 68.43% of total cells in Pa. For purple sea cucumbers in the late pigmentation stage, clusters 0, 1, 2, 4, and 5 were the largest, accounting for 67.38% of total cells in Pc. For green sea cucumbers in the early pigmentation stage, clusters 0, 1, 2, 3, and 4 were the largest, accounting for 73.14% of total cells in Ga. For green sea cucumbers in the late pigmentation stage, clusters 0, 1, 2, 3, and 7 were the largest, accounting for 64.70% of total cells in Gc (Table S5).

### 2.2. Identification of Cell Populations

To characterize cell clusters present in the datasets, we automatically annotated these cell clusters using SingleR. Figure S1A shows the cell annotation results according to the human genome, and Figure S1B shows the cell annotation results according to the mouse genome. As shown in Figure S1A, clusters 0, 6, 8, 11–15, and 17–25 belonged to epithelial cells; clusters 1–5 and 10 belonged to tissue stem cells; cluster 7 belonged to monocytes; clusters 9 and 16 belonged to neutrophils. As shown in Figure S1B, clusters 0–5, 8–15, and 17−24 belonged to fibroblasts; clusters 6 and 25 belonged to neurons; cluster 7 belonged to endothelial cells; cluster 16 belonged to endothelial cells.

**Figure 2:**
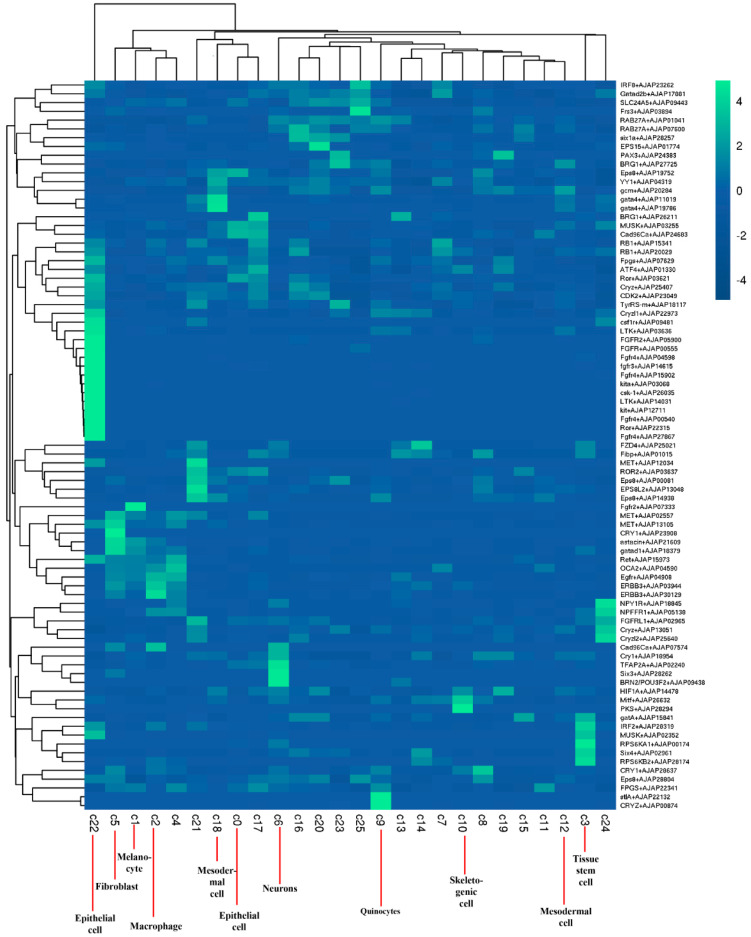
Identification of sea cucumber body-wall cell populations. Heatmap of curated genes selected as cell markers.

SingleR annotation results are not completely accurate and can only be used as an auxiliary reference. Therefore, differential gene expression analysis was performed across the 26 clusters. Dot plots of the top upregulated genes from each cluster are shown in Figure S2. Using enriched curated marker gene expression, we could assign cell types or cell type precursors to 11 of the 26 different clusters (Figure 2, Table S6). Cluster 9 was a pigment cell population, with enriched expression of polyketide synthase1 (PKS1) and quinone oxidoreductase. Cluster 10 corresponded to skeletogenic cells highly expressing polyketide synthase2 (PKS2). Clusters 12 and 18 expressing gataE and GCM were clustered into mesodermal cells. Cluster 5 with expression of astacin belonged to fibroblasts. Cluster 1 was a melanocyte cell population, with enriched expression of melanotransferrin. Cluster 6 corresponded to neurons highly expressing embryonic lethal abnormal visual system (Elav). Cluster 2 expressing CD163 belonged to macrophages. Cluster 3 was a tissue stem-cell population, with enriched expression of Cyclin-L1 and a gene with a trinucleotide repeat (TNR). Lastly, clusters 0 and 22 expressing fibroblast growth factor receptor (FGFR) were clustered into epithelial cells.

### 2.3. Pseudotime Analysis

**Figure 3:**
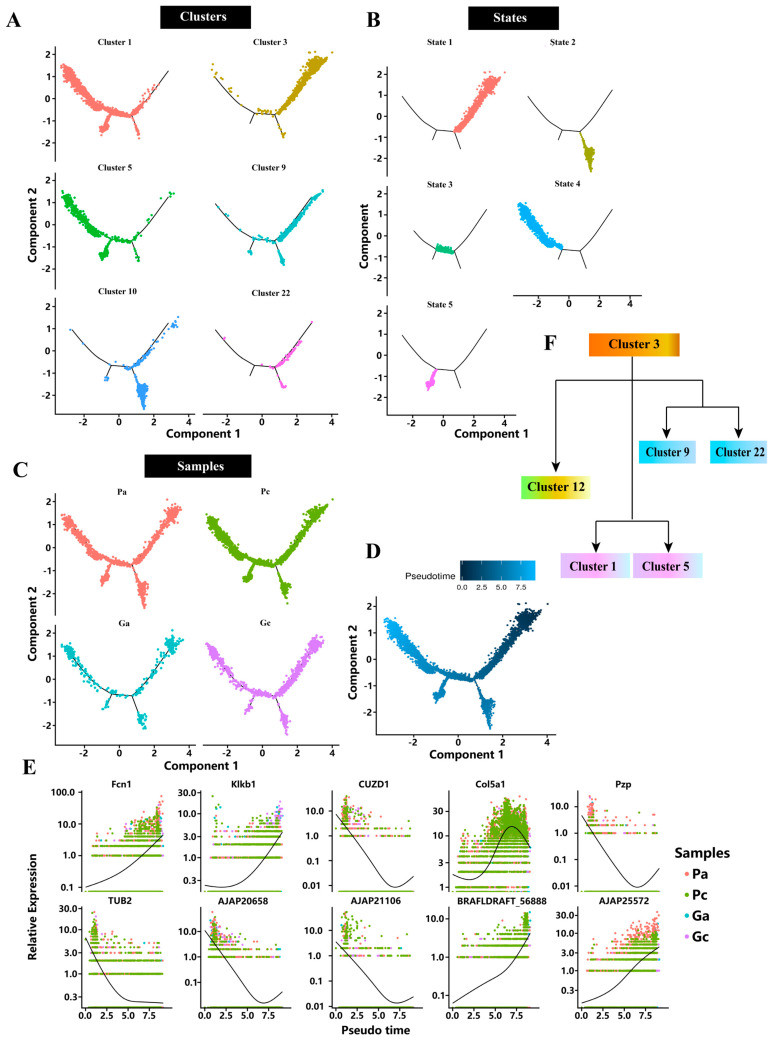
Pseudotime analysis of cluster 1, cluster 3, cluster 5, cluster 9, cluster 10, and cluster 22. (**A**) Cell trajectory distribution of different clusters. (**B**) Cell trajectory distribution of different states. (**C**) Cell trajectory distribution of different samples. (**D**) Cell trajectory pseudotime distribution map. Different points represent different cells. The darker the point color is, the earlier the development period is. (**E**) Pseudotime analysis of top 10 differentially expressed genes. The black solid line is the fitting line of the gene expression level, and the different colors represent the samples. (**F**) Differentiation relationship diagram of cluster 1, cluster 3, cluster 5, cluster 9, cluster 10, and cluster 22.

We performed pseudotime analysis to elucidate the transcriptome dynamics of selected key cell clusters during the pigmentation process in sea cucumber. The discriminative dimensionality reduction tree (DDRTree) algorithm was used to determine the pseudo-developmental time and to map the developmental trajectory (Figure 3A–D). It is well known that pigments and pigment cells are key factors determining the formation of body color. On the basis of marker genes of pigment cells in sea urchins, we identified cluster 9 as pigment cells in sea cucumbers. A previous study showed that melanin is a key pigment in the formation of sea cucumber body color, which is mainly related to the density of mature melanosomes and the amount of melanin granules secreted from melanosomes [12]. Here, we identified cluster 1 as melanocytes in sea cucumber. Then, we wanted to investigate the developmental sequence of cells expressing quinone pigments (quinocytes) and cells expressing melanin (melanocytes) in sea cucumbers. For this, we subjected the identified melanocytes, tissue stem cells, fibroblasts, quinocytes, skeletogenic cells, and epithelial cells to pseudotime analysis. The results showed that tissue stem cells were involved in the earliest stage of development, followed by quinocytes and epithelial cells. Skeletogenic cells developed at a slightly later stage, and melanocytes and fibroblasts were involved in the latest stage of development (Figure 3). In addition, from the pseudotime analysis results, we could see that the developmental states of quinocytes and epithelial cells were similar, while the developmental states of melanocytes and fibroblasts were similar.

On the basis of the pseudotime values for each cell, DEGs along the pseudotime axis were screened, and cluster analysis was conducted on DEGs to identify genes with similar expression trends. Meanwhile, a trajectory diagram of the expression levels of DEGs along the pseudotime axis was drawn according to the significance. For example, expression levels of Fcn1 and Klkb1 increased gradually, while expression of TUB2 decreased during the pigmentation process. Expression of the COL5A1 gene went from high to low, while that of the Pzp gene went from low to high.

### 2.4. Analysis of DEGs at Different Development Stages

To characterize the pigmentation process in green and purple sea cucumbers, we determined DEGs between cell populations in Ga vs. Gc and Pa vs. Pc comparison groups. Compared with green sea cucumbers in the early pigmentation stage, DEGs of green sea cucumbers in the late pigmentation stage were mainly found in clusters 0, 2, 1, 4, and 3. Compared with purple sea cucumbers in the early pigmentation stage, DEGs in purple sea cucumbers in the late pigmentation stage were mainly found in clusters 0, 1, 5, 4, and 2 (Table S7). Here, we focused on cluster 1 melanocytes and cluster 9 quinocytes.

**Figure 4:**
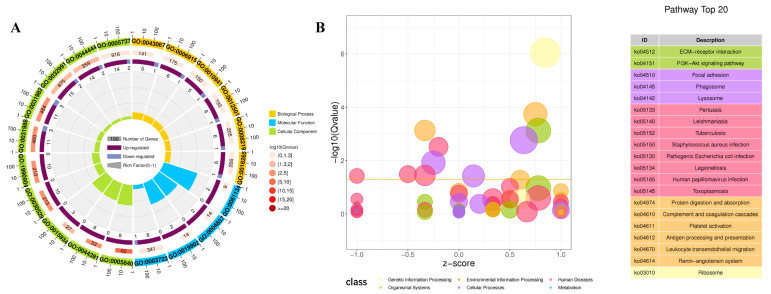
GO and KEGG analysis of cluster 1 in Ga vs. Gc comparison group. (**A**) The top 20 most significant GO terms. (**B**) The top 20 most significant pathways.

DEGs in cluster 1 from the Ga vs. Gc comparison group were significantly enriched in GO terms related to ribosome, vesicle, apoptotic process, macromolecular complex, peptidase regulator activity, cytoplasmic part, and serine family amino-acid metabolic process (Figure 4A). Significantly enriched pathways of DEGs in cluster 1 of the Ga vs. Gc comparison group included extracellular matrix (ECM) receptor interaction, focal adhesion, PI3K/Akt signaling pathway, phagosome, lysosome, and protein digestion and absorption (Figure 4B). DEGs in cluster 1 of the Pa vs. Pc comparison group were significantly enriched in GO terms related to ribosome, ribonucleoprotein complex, heterocyclic compound binding, cell–substrate junction, cytoplasmic vesicle, and protein folding (Figure S3A), and related pathways were protein processing in endoplasmic reticulum, oxidative phosphorylation, lysosome, thermogenesis, protein digestion and absorption, citrate/tricarboxylic acid (TCA) cycle, ECM receptor interaction, and focal adhesion (Figure S3B). DEGs in cluster 9 of the Pa vs. Pc comparison group were significantly enriched in GO terms related to ribosome, TCA metabolic process, amide biosynthetic process, heterocyclic compound binding, macromolecular complex, peptide biosynthetic process, amide biosynthetic process, regulation of translation, electron carrier activity, and organic cyclic compound binding (Figure S4A). Significantly enriched pathways for DEGs in cluster 9 of the Ga vs. Gc comparison group included ribosome, oxidative phosphorylation, propanoate metabolism, pyruvate metabolism, fatty-acid metabolism, tyrosine metabolism, AMPK signaling pathway, and tryptophan metabolism (Figure S4B).

In addition, significantly expressed genes in cluster 1 and 9 of the Ga vs. Gc and Pa vs. Pc comparison groups are shown in Table S8. Collagen type II alpha-1 gene (COL2A1) was significantly upregulated in cluster 1 of the Ga vs. Gc and Pa vs. Pc comparison group. The expression levels of the solute carrier family 25 member 5 (SLC25A5) were upregulated in the Ga vs. Gc comparison group and downregulated in the Pa vs. Pc comparison group. Zinc finger protein 36-like 1 (ZFP36L1) was significantly upregulated in cluster 1 of the Ga vs. Gc comparison group. SVEP1 was significantly upregulated in cluster 1 of the Ga vs. Gc and Pa vs. Pc comparison groups (Table S8).

### 2.5. Analysis of DEGs in Different Color Morphs

To characterize color polymorphism in sea cucumber, we determined DEGs between cell populations in Ga vs. Pa and Gc vs. Pc comparison groups. Compared with green sea cucumbers in the early pigmentation stage, DEGs in purple sea cucumbers in the early pigmentation stage were mainly found in clusters 0, 1, 2, 4, and 3. Compared with green sea cucumbers in the late pigmentation stage, DEGs in purple sea cucumbers in the late pigmentation stage were also mainly found in clusters 0, 1, 2, 3, and 4 (Table S7).

DEGs in cluster 1 of the Ga vs. Pa comparison group were significantly enriched in GO terms related to ribosomes, macromolecular complexes, cell junctions, macromolecule metabolic processes, co-translational proteins targeting membranes, and viral processes (Figure S5A). Significantly enriched pathways of DEGs in cluster 1 of the Ga vs. Pa comparison group included ribosomes, lysosomes, glycosaminoglycan degradation, protein digestion and absorption, ECM receptor interaction, Th17 cell differentiation, phagosomes, PI3K/Akt signaling pathways, and fatty-acid biosynthesis (Figure S5B). DEGs in cluster 1 of the Gc vs. Pc comparison group included genes enriched in GO terms related to contractile fibers, vesicles, glucose homeostasis, regulation of cell growth, myofibrils, peptidase activator activity, and regulation of protein stability (Figure S6A); the associated pathways were lysosomes, ribosomes, focal adhesion complexes, phagosomes, leukocyte transendothelial migration, glycosaminoglycan degradation, protein digestion and absorption, regulation of actin cytoskeleton, protein processing in endoplasmic reticulum, and Rap1 signaling (Figure S6B). DEGs in cluster 9 of the Gc vs. Pc comparison group were significantly enriched in GO terms related to phosphatase binding, peptidase activator activity, positive regulation of cysteine-type endopeptidase activity involved in apoptotic process, positive regulation of proteolysis, regulation of endopeptidase activity, contractile fiber, and negative regulation of transferase activity (Figure S7A). Significantly enriched pathways of DEGs in cluster 9 of the Gc vs. Pc comparison group included ribosomes, protein processing in the endoplasmic reticulum, the citrate (TCA) cycle, Jak/STAT signaling, Th17 cell differentiation, and Th1 and Th2 cell differentiation (Figure S7B).

Like the Ga vs. Gc and Pa vs. Pc comparison groups, COL2A1 was also significantly upregulated in cluster 1 of the Ga vs. Pa and Gc vs. Pc comparison group, suggesting that expression of the COL2A1 gene in purple sea cucumber was higher than in green sea cucumber. Regarding SLC25A5, this was significantly upregulated in cluster 1 of the Ga vs. Pa comparison group and significantly downregulated in cluster 1 of the Gc vs. Pc comparison group. Expression levels of ZFP36L1 were not significantly different between green and purple sea cucumbers during two pigmentation stages. Meanwhile, expression levels of SVEP1 were higher in purple sea cucumbers than in green sea cucumbers during both pigmentation stages. The ferritin gene was significantly upregulated in the Ga vs. Pa and Gc vs. Pc comparison groups. Furthermore, significantly expressed genes in cluster 1 and 9 of the Ga vs. Pa and Gc vs. Pc comparison groups are shown in Table S8.

### 2.6. Melanin and Quinone Pigments Content in Sea Cucumbers

**Figure 5:**
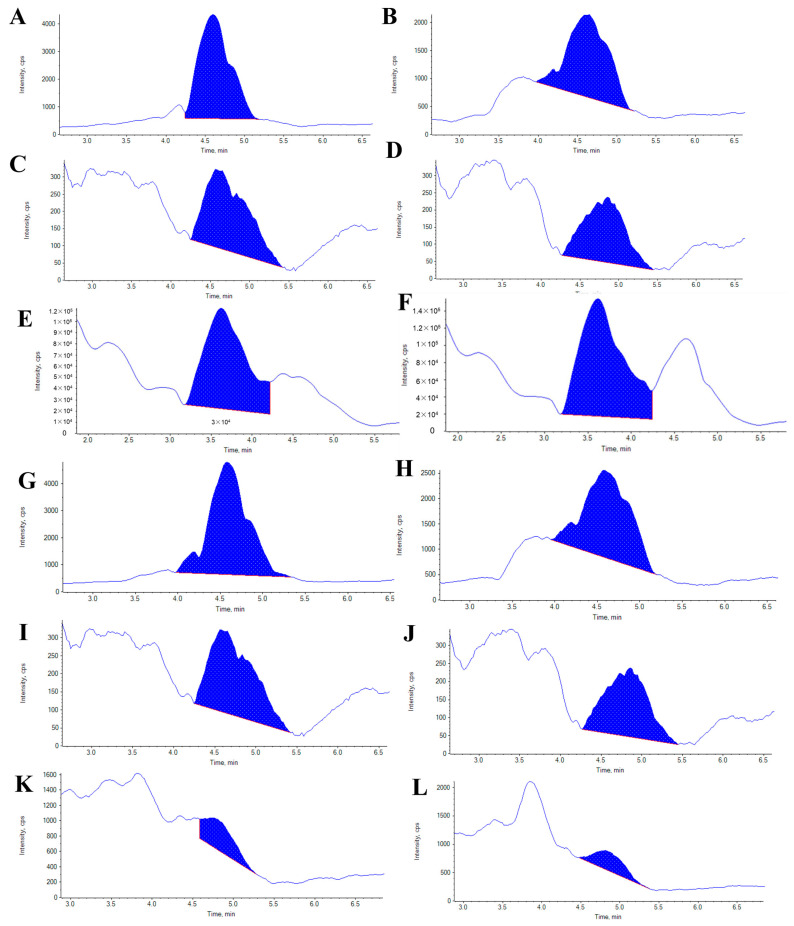
LC–MS analysis of melanin and quinone pigments extracted from body walls of purple and green sea cucumbers. (**A**,**C**,**E**,**G**,**I**,**K**) Melanin, chimaphilin, menadione, juglone, lawsone, and 5,8-dihydroxy-1, 4-naphthoquinone in purple sea cucumber, respectively. (**B**,**D**,**F**,**H**,**J**,**L**) Melanin, chimaphilin, menadione, juglone, lawsone and 5,8-dihydroxy-1, and 4-naphthoquinone in green sea cucumber, respectively.

We analyzed the differences in the contents of melanin and five quinone pigments in the body wall of the purple and the green sea cucumber, and the results showed that the contents of melanin and quinone pigments in the purple sea cucumber were all higher than those in the green sea cucumber (Table 1). Figure 5 shows the integration diagram of the six pigments targeting analyses obtained by the MultiQuant Algorithm. Melanin, with dark appearance, is formed by the polymerization of phenolic and indolic compounds [13]. It serves diverse functions that range from camouflage and protection to energy harvesting. Melanin played an important role in body color formation in the purple and green sea cucumbers, with concentrations of 41.21 and 19.12 µg·g^−1^, respectively. The melanin content in the purple sea cucumbers was 115.5% higher than in the green ones. Naphthoquinone pigments are the most commonly occurring type of quinones in nature, which have antibacterial, anti-inflammatory, neuroprotective, dyeing, and antitumor properties. Here, we detected five naphthoquinone pigments in the body wall of sea cucumbers: chimaphilin (2,7-dimethyl-1,4-naphthoquinone), menadione (2-methyl-1,4-naphthoquinone, vitamin K3), juglone (5-hydroxy-1,4-naphthoquinone), lawsone (2-hydroxy-1,4-naphthoquinone), and 5,8-dihydroxy-1,4-naphthoquinone. Chimaphilin and menadione and were found to be high in both color morphs of sea cucumbers. These two pigments were present in purple sea cucumbers in contents of 57.88 and 40.33 µg·g^−1^ and in green sea cucumbers in contents of 31.26 and 53.15 µg·g^−1^, respectively. Juglone had a low concentration in sea cucumbers; it was 10.15 µg·g^−1^ in purple sea cucumber and 1.32 µg·g^−1^ in green sea cucumber, respectively. In addition, the content of lawsone in green sea cucumber was 8.23 µg·g^−1^, while the content of this pigment in purple sea cucumber was more than three times of that in green sea cucumber. The contents of 5,8-dihydroxy-1,4-naphthoquinone in purple sea cucumber and green sea cucumber were 23.68 and 16.2 µg·g^−1^, respectively. With the exception of menadione, purple sea cucumbers had higher contents of the other four quinone pigments and melanin than green sea cucumbers did.

## 3. Discussion

Single-cell sequencing technology is useful for detailed analysis of cell types and gene expression patterns for many complex tissues. Herein, single-cell sequencing of body-wall cells from green and purple sea cucumbers in two pigmentation stages was performed to investigate the transcriptional trajectories and heterogeneity of body color formation, including cell classification and functional differences. Our results showed that pigment cells from the purple and green sea cucumbers expressed a distinct set of genes at different levels, consistent with the premise that these genes function in the color morphology of the animals. Structurally, the body wall of sea cucumbers consists of a pigmented layer of the epidermis, dermal connective tissues, and inner muscular layers [14]. The epidermis is the outermost layer of the body and protects it from environmental insults. In the present study, we identified epithelial, melanocyte, quinocytes, fibroblast, skeletogenic, tissue stem cells, neurons, and macrophage cells in the body wall of sea cucumbers. Melanocytes and quinocytes are responsible for the synthesis of melanin and quinone pigments, respectively, and play a key role in the pigmentation of sea cucumbers, which is worthy of further investigation. With candidate genes for each of the major pigment types and pigment bearing cells, we can now systematically test function through the judicious use of Cas9 targeting of select pigment-related genes with the outcome in the animals both by visualization and by MS analysis.

Melanocytes are differentiated from melanocyte progenitors, which in vertebrates originate from neural crest cells in early development [15]. Neural crest cells are multipotent cells that can differentiate into neurons, glia, adrenal medulla, fibroblasts, and melanocytes, and these cells are vertebrate-specific pluripotent stem cells [16]. They arise from the dorsal aspect of the neural tube, migrate to specific tissue targets along defined paths, and differentiate into a variety of cells [17,18,19]. In zebrafish, melanocytes arise from a small number of bipotent melanogenic progenitors that are derived directly from the neural crest without a stem-cell intermediate during the first 3 days of development, and from another population that arises via a melanocyte stem-cell intermediate [20,21,22]. The melanocyte lineage of neural crest contains melanosomes, lysosome-related organelles in which melanins are synthesized and stored [23]. Melanin has many functions [24], such as scavenging reactive free radicals [25], enhancing immunity [26,27], resistance to radiation, aging, and oxidation [28,29,30,31], and strengthening the body structure [32]. In the present study, we found tissue stem cells and melanocytes in the body wall of sea cucumbers that had many characteristics of the melanocytes of neural crest origins.

On the basis of the results of pseudotime analysis, we hypothesized that melanocytes may be derived from tissue stem cells in echinoderms. Furthermore, sea cucumbers had differentiated melanocytes at the early pigmentation stage or even earlier. However, in the early pigmentation stage there were no mature melanosomes in melanocytes, resulting in no melanin production. As pigmentation progresses, melanosomes mature and synthesize melanin to play a key role in pigmentation and body color formation. Here, we found that the melanin content in purple sea cucumber was significantly higher than in green sea cucumber. We hypothesize that this is due to the higher number and proportion of melanocytes in purple sea cucumbers than in green sea cucumbers on the one hand, and the upregulation of COL2A1 and other key genes on the other.

COL2A1 is a critical secreted regulator of melanogenesis and epidermal pigmentation. SLC25A5 has been shown to biologically affect pigmentation [33,34], and variants of SLC25A5 are associated with skin color in human [35]. ZFP36L1, a CCCH-type zinc finger protein, is a potent regulator of keratinocyte vascular endothelial growth factor (VEGF) production. It regulates the production of growth factors and cytokines via destabilization of the respective mRNAs [36,37]. SVEP1, also known as Polydom, is a large extracellular mosaic protein that functions in protein interactions and adhesion [38]. A previous study indicated that SVEP1 plays a critical role during epidermal differentiation [39]. Ferritin has been explored as a novel and natural strategy for iron supplementation [40], and it is located within neuromelanin granules, an iron storage organelle in dopaminergic neurons of the human brain [41]. These genes may contribute to the color morph differentiation of sea cucumbers and will be tested for pigmentation function in the future by CRISPR/Cas9 approaches.

Echinoderms display a vast array of pigmentation and patterning in larval and adult life stages. This coloration is thought to be important for immune defense and camouflage. Sea urchin larvae are pigmented due to the accumulation of a red/orange pigment in single cells [42]. This pigment is a napthoquinone called echinochrome A, which accumulates in pigment cell precursors [43,44]. Adult sea urchins that lack pigments are less resistant to environmental challenges [45]. The developmental origins of pigment cells in purple sea urchin have been traced to a group of mesodermal cells, the non-skeletogenic mesoderm (NSM) [46,47,48]. A recent study found two distinct populations of pigment cells in sea urchins [9], and two hypotheses were proposed to explain the finding of a mitotic pigment cell cluster; either there is a unique stem-cell population of pigment cells, or all pigment cells have the ability to divide [9]. Here, we identified pigment cells (quinocytes) in the sea cucumber body wall using marker genes in sea urchin pigment cells. During the process of pigmentation, the number and proportion of pigment cells increased significantly in both green and purple sea cucumber, but there were significantly more pigment cells in the purple color morph. Correspondingly, the content of quinone pigments in purple sea cucumber was also higher than that in green sea cucumbers.

Importantly, here we were able to link gene activity and expression with the pigment products found in those cells. We reported five naphthoquinone pigments present in the body wall of sea cucumbers. Chimaphilin, 2,7-dimethyl-1,4-naphthoquinone, is a yellow naphthoquinone which occurs naturally in various Chimaphila and Pyrola species [49]. It possesses highly efficient antitumor, antifungal, and antioxidant activities [50]. Menadione, known as an antitumor factor, is sometimes used as a dietary supplement for its vitamin K property and is, hence, called vitamin K3 [51]. Kim et al. found that melanin content was significantly reduced after menadione treatment in a dose-dependent manner [52]. In this study, we discovered that the content of menadione in green sea cucumber was significantly higher than that in the purple color morph, which may be one of the explanations for the lower melanin content in the green color morph than in the purple color morph. Juglone is a natural pigment, which has a cytotoxic effect against various human tumor cells [53]. Studies have demonstrated that juglone exhibits anticancer, antibacterial, antiviral, sedative, and antihypertensive properties [54,55]. Furthermore, it is widely used as a reddish-brown natural dye [56]; thus, it is key that we now see the cell’s repertoire of gene activities to help explain the biosynthesis of this key drug potential. Lawsone (2-hydroxy-1,4-naphthoquinone) is a natural naphthoquinone present in the henna leaf extract with several cytotoxic activities and used as precursor for synthesis of various pharmaceutical compounds [57]. 5,8-Dihydroxy-1,4-naphthoquinone is a naphthoquinone derivative with important antibacterial activity [58], and it is also one of the key chromophores in cellulosic materials [59]. Naphthoquinone pigments have various physiological functions such as antibacterial, cardioprotective, and antioxidant. Many naphthoquinone derivatives also possess antitumor activity in vitro [60,61]. Hence, the naphthoquinone pigments from sea cucumbers have good medical perspectives as anticancer and antiviral preparations. Given the clear benefit of naphthoquinone pigments in purple sea cucumbers compared to green sea cucumbers, we may focus on the body color formation of purple sea cucumber, as well as use it as a significant research tool for anticancer and antivirus research in the future.

## 4. Materials and Methods

### 4.1. Animal Sampling

Green and purple sea cucumber populations were obtained by mating appropriately colored females and males in March 2019. For example, green sea cucumber populations were obtained by mating green females with green males. The parents of each color morph used in this experiment were supplied by Shandong Oriental Ocean Sci-Tech Co., Ltd. (Yantai City, Shandong Province, China). Rearing of embryos, larvae, and juveniles was carried out using standard practices [62]. In addition to developmental timing, selection of samples for analysis were also based on individual appearance. During the early pigmentation stage, the body of *A. japonicus* is transparent, and the intestine and other organs can be clearly seen. During the late pigmentation stage, *A. japonicus* transitions to an opaque body color, and both dorsal and ventral surfaces are pigmented. Here, three green sea cucumbers in the early pigmentation stage (Ga) and three purple sea cucumbers in the early pigmentation stage (Pa) were selected at 15 days after fertilization (DAF). Three green sea cucumbers in the late pigmentation stage (Gc) and three purple sea cucumbers in the late pigmentation stage (Pc) were selected at 30 DAF. The body wall of these experimental animals was sampled for subsequent molecular and biochemical experiments.

### 4.2. Body-Wall Dissociation and Single-Cell Suspension Preparation

Body-wall samples were cut into small fragments and transferred to centrifuge tubes; then, 1 mL of enzyme mixture (0.125% collagenase type IA in calcium and magnesium free artificial sea water solution (CMFSS)) was added for 45 min at 28 °C. The tissues were dissociated by shear force with a pipette, and, after filtering through a 70 μm mesh, the dissociations were centrifuged for 1 min at 300× *g*, 4 °C (Eppendorf, Hamburg, Germany). The supernatant was aspirated, and a 5 mL volume of precooled CMFSS was added to the pellet, which was gently pipetted five times with a wide-bore pipette tip to resuspend the cells. Pelleting of the cells was then repeated, and the resuspension was filtered through a 40 μm mesh. The cells were placed on ice and used for analysis by 10× Genomics within 30 min.

### 4.3. Library Preparation and Sequencing

Cell viability and counting were evaluated by Trypan Blue staining using a Countess II Automated Cell Counter (Thermofisher, Waltham, MA, USA), and samples with viability >80% were used for sequencing. Single-cell encapsulation was performed using the Chromium Single-Cell Chip B Kit and a 10× Genomics Chromium Controller (10× Genomics, Pleasanton, CA, USA). Single-cell cDNAs and libraries were prepared according to the manufacturer’s protocol using a Chromium Single Cell 3′ Reagent Kit v3 (10× Genomics, Pleasanton, CA, USA). Libraries were sequenced on an Illumina sequencing platform by Genedenovo Biotechnology Co., Ltd. (Guangzhou, China).

### 4.4. Quality Control and Data Processing

The aligner STAR tool (https://github.com/alexdobin/STAR (accessed on 21 November 2022)) from Cell Ranger (http://support.10xgenomics.com/single-cell/software/overview/welcome (accessed on 21 November 2022)) was used to perform splicing-aware alignment of reads to the genome (http://www.genedatabase.cn/aja_genome_20161129.html (accessed on 21 November 2022)). Cell Ranger then used transcript annotation GTF files to group reads into exonic, intronic, and intergenic, according to whether reads aligned to the *A. japonicus* genome. A read is exonic if at least 50% of it intersects an exon, intronic if it is non-exonic and intersects an intron, and intergenic otherwise. A read that is compatible with the exons of an annotated transcript, aligned to the same strand, is considered mapped to the transcriptome. If a read is compatible with a single gene annotation, it is considered uniquely mapped to the transcriptome. Only reads that are uniquely mapped to the transcriptome are used for unique molecular identifier (UMI) counting.

Cells were then filtered by the following criteria: cells with <200 genes or >3000 genes detected, cells with >8000 UMIs detected, and cells for which >10% of UMIs were derived from mitochondrial genes were excluded. After removing low-quality cells from the dataset, we employed the ‘LogNormalize’ global-scaling normalization method that normalizes gene expression measurements for each cell against total expression, multiplies this by a scale factor (10,000 by default), and log-transforms the result. The formula is as follows:

Gene expression level = log (1 + [UMIA / UMI total] × 10,000).

We then conducted batch effect correction by canonical correlation analysis. We implemented a resampling test inspired by the jackStraw procedure [63]. We randomly permuted a subset of the data (1% by default) and reran the principal component analysis (PCA), constructing a ‘null distribution’ of gene scores, and we repeated this procedure to yield the single-cell datasets.

### 4.5. t-Distribution Stochastic Neighbor Embedding (t-SNE) Visualization and SingleR Analysis

Distances between cells were calculated on the basis of previously identified principal components. Cells were embedded in a K-nearest neighbor (KNN) graph on the basis of the Euclidean distance between cells. The KNN map was partitioned into highly interconnected quasi-cliques according to the Jaccard distance between the two cells locally adjacent. The Louvain algorithm was applied to iteratively group cells together, with the goal of optimizing the standard modularity function. Next, t-SNE [64] was used as a powerful tool to visualize and explore these datasets. t-SNE aims to place cells with similar local environments in high-dimensional space together in low-dimensional space. t-SNE projection and clustering analysis for all datasets were conducted using 50 dimensions and a resolution of 0.5.

SingleR is an R package for automatic cell type annotation of scRNA-seq data [65]. It annotates cells in test data that are similar to the reference set using a given cell sample with a known type of tag as the reference dataset. Specifically, we calculated the Spearman correlation between the expression profile of each cell and the expression profile of the reference sample. This was achieved by combining marker genes identified between all marker pairs. We then defined the score of each label as a fixed quantile of the relevant distribution (default 0.8). Finally, we repeated this operation for all labels, then used the label with the highest score as the annotation for this cell.

### 4.6. Analysis of Differentially Expressed Genes (DEGs)

Seurat [66] was used for analysis of DEGs. New idents were set as ‘group_cluster’ or ‘group_cell type’ for analysis [67]. We then used a hurdle model in MAST (Model-Based Analysis of Single-Cell Transcriptomics) [68] to find DEGs for a group in one cluster. We identified DEGs using the following criteria: (1) |log_2_FC| ≥ 1; (2) adjusted *p*-value ≤ 0.01; (3) percentage of cells where the gene is detected in specific cluster > 25%.

Gene Ontology (GO) enrichment analysis provides all GO terms significantly enriched in peak-related genes compared with the genome background, and filters peak-related genes that correspond to biological functions. Firstly, all peak-related genes were mapped to GO terms in the GO database (http://www.geneontology.org/ (accessed on 21 November 2022)), gene numbers were calculated for every term, and significantly enriched GO terms of peak-related genes compared with the genome background were defined by hypergeometric tests.

Gene products usually interact with each other to fulfil certain biological functions, and pathway-based analysis helps to further understand the biological functions of genes. Kyoto Encyclopedia of Genes and Genomes (KEGG) is the major public pathway-related database [69]. Pathway enrichment analysis identified significantly enriched metabolic pathways or signal transduction pathways of peak-related genes compared with the whole-genome background. The calculated *p*-values were subjected to false discovery rate (FDR) correction, with FDR ≤ 0.05 as a threshold of confidence. GO terms meeting this condition were defined as significantly enriched GO terms of peak-related genes. Pathways meeting this condition were defined as significantly enriched pathways of peak-related genes.

### 4.7. Pseudotime Analysis

Pseudotime analysis, also known as cell trajectory analysis, can be used to infer the differentiation trajectory of cells or the evolution process of cell subtypes in the course of development. Here, we used Monocle [70] to conduct pseudotime analysis. Monocle can, in principle, be used to recover single-cell gene expression kinetics from a wide array of cellular processes, including differentiation, proliferation, and oncogenic transformation [70]. The cell gene expression matrix was transferred into Monocle, and Monocle was applied to reduce the dimensionality of the data to a two-dimensional plane. Cells were arranged into a tree structure with branches and nodes; then, according to biological significance, the cell population with the most primitive differentiation state was defined as the cell population with the smallest pseudotime in the cell trajectory, and the pseudotime value of all cells was calculated.

On the basis of the gene expression levels of cells in various differentiated states, genes differentially expressed in differentiated states were screened using FDR < 10^−7^ as a cutoff. On the basis of the pseudotime value, Monocle modeled gene expression levels as a smooth and nonlinear function to explore gene expression changes with pseudotime values. We screened genes with FDR < 10^−7^ as differentially expressed genes. When differentiation fate choice occurs in the process of development, there will be branching in the cell trajectory, and the cell will follow two different developmental lineages. In order to identify genes with different expression patterns in the two branches, negative binomial generalized linear models were fitted for the two branches, and differential genes associated with different branches were found and tested by comparing the two models.

### 4.8. Pigment Content Analysis

Samples of sea cucumber body wall were ground under liquid nitrogen, and the ground samples were placed in 2 mL centrifuge tubes. The samples were lyophilized, and 100 milligrams of each sample was placed into a new 2 mL centrifuge tube, to which 600 μL of methanol and 200 μL of chloroform were added. The samples were then placed in a high-throughput tissue grinder under the condition of grinding at 65 Hz for 90 s. The milled samples were vortexed for 2 min, followed by sonication at 4 °C for 30 min. After that, the samples were centrifuged in a cryogenic centrifuge at 4 °C for 15 min at 12,000 rpm. After centrifugation, the supernatant was extracted for liquid chromatography/mass spectrometry (LC–MS) analysis. The mobile phase was water and methanol, and the flow rate was 0.3 mL/min. The specific parameters of each pigment are shown in Table S9.

## 5. Conclusions

In conclusion, we identified the transcriptome profile of two key cell groups responsible for the formation of sea cucumber body color by scRNA-seq: melanocytes and quinocytes. These two cell groups in purple sea cucumber were more abundant than in green sea cucumber, consistent with the higher content of melanin and quinone pigment in the purple color morph. In addition, important genes related to pigmentation were identified, including COL2A1, SLC25A5, SVEP1, PKS1, and GCM. This study expands our understanding of body color formation in sea cucumbers and provides support for the development and application of bioactive substances of sea cucumber.

## Figures and Tables

**Table 1:** Melanin and quinone pigments in purple and green sea cucumbers (mean ± SD; µg per g).

Pigment	Purple *A. japonicus*	Green *A. japonicus*
Melanin	41.21± 1.22	19.12 ± 1.11
Chimaphilin (2,7-dimethyl-1,4-naphthoquinone)	57.88 ± 4.98	31.26 ± 1.53
Menadione (2-methyl-1,4-naphthoquinone)	40.33 ± 0.39	53.15 ± 1.95
Juglone (5-hydroxy-1,4-naphthoquinone)	10.15 ± 1.12	1.32 ± 0.17
Lawsone (2-hydroxy-1,4-naphthoquinone)	27.69 ± 1.43	8.23 ± 0.57
5,8-dihydroxy-1,4-naphthoquinone	23.68 ± 2.63	16.20 ± 1.31

## Data Availability

Raw scRNA-seq data were deposited in the Oceanographic Data Center, Chinese Academy of Sciences (CASODC; http://msdc.qdio.ac.cn (accessed on 21 November 2022)) and are publicly accessible at http://dx.doi.org/10.12157/IOCAS.20220406.001 (accessed on 21 November 2022).

## Supplementary Materials

The following supporting information can be downloaded at https://www.mdpi.com/article/10.3390/ijms241512213/s1. Figure S1: Cell annotation by SingleR. (A) Human database; (B) Mouse database; Figure S2: The Dotplot of top up-regulated expression genes from each cluster; Figure S3: GO and KEGG analysis of cluster 1 in Pa Vs Pc comparison group. (A) The top 20 most significant GO terms; (B) The top 20 most significant pathways; Figure S4: GO and KEGG analysis of cluster 9 in Pa Vs Pc comparison group. (A) The top 20 most significant GO terms; (B) The top 20 most significant pathways; Figure S5: GO and KEGG analysis of cluster 1 in Ga Vs Pa comparison group. (A) The top 20 most sig-nificant GO terms; (B) The top 20 most significant pathways; Figure S6: GO and KEGG analysis of cluster 1 in Gc Vs Pc comparison group. (A) The top 20 most significant GO terms; (B) The top 20 most significant pathways; Figure S7: GO and KEGG analysis of cluster 9 in Gc Vs Pc comparison group. (A) The top 20 most significant GO terms; (B) The top 20 most significant pathways; Table S1: The sample sequencing quality; Table S2: Reads statistics of each sample; Table S3: Cells sta-tistics in each sample before and after filtering; Table S4: Statistics of cell clusters; Table S5: Cell number and ratio of each sample in different cluster; Table S6: Cell cluster annotations with the most representative marker genes; Table S7: Differentially expressed genes (DEGs) in different comparison groups; Table S8: significantly expressed genes in cluster 1 and 9 of the Ga vs. Pa and Gc vs. Pc comparison groups; Table S9: Acquisition parameters of melanin and quinone pigments.
