# Supplementary material for: Single-Cell Transcriptome and Pigment Biochemistry Analysis Reveals the Potential for the High Nutritional and Medicinal Value of Purple Sea Cucumbers
Source: Int J Mol Sci. 2023 Jul 30;24(15):12213. doi: 10.3390/ijms241512213 (PMC10419132; doi:10.3390/ijms241512213)
Supplement: Supplementary file 1 [file ijms-24-12213-s001.zip › Supplementary Tables S1-S7 and S9.pdf]

Table S1 The sample sequencing quality.

Sample	Number of Reads	Valid Barcodes	Sequencing Saturation	Q30 Bases in Barcode	Q30 Bases in RNA Read	Q30 Bases in UMI
Pa	472,616,332	94.30%	79.10%	93.60%	88.40%	91.40%
Pc	528,581,234	94.20%	79.90%	93.90%	88.80%	91.60%
Ga	412,137,914	95.10%	79.30%	93.50%	87.50%	91.60%
Gc	491,872,382	95.10%	72.70%	93.90%	88.10%	91.60%

Table S2 Reads statistics of each sample.

Sample	Estimated Number of Cells	Fraction Reads in Cells	Mean Reads per Cell	Median Genes per Cell	Total Genes Detected	Median UMI Counts per Cell	Reads Mapped Confidently to Genome	Reads Mapped Confidently to Intergenic Regions	Reads Mapped Confidently to Intronic Regions	Reads Mapped Confidently to Exonic Regions	Reads Mapped Confidently to Transcriptome
Pa	8,010	37.30%	59,003	603	27,842	1,090	61.30%	14.60%	2.50%	44.20%	34.40%
Pc	8,946	42.20%	59,085	573	28,084	1,026	61.10%	15.70%	2.70%	42.70%	32.40%
Ga	7,100	58.50%	58,047	842	27,407	1,535	64.50%	15.50%	3.00%	46.00%	36.70%
Gc	7,953	37.80%	61,847	768	28,063	1,420	63.30%	15.10%	3.40%	44.80%	35.10%

Table S3 Cells statistics in each sample before and after filtering.

Sample	Before filter num	After filter num	percentage	After filter median UMI/cell	After filter median genes/ cell
Pa	8010	7915	98.81%	1079	600
Pc	8946	8839	98.80%	1001	562
Ga	7100	6871	96.77%	1286	733
Gc	7953	7818	98.30%	1367	745

Table S4 Statistics of cell clusters.

Cluster	Cells number	Portion	Median Genes per Cell	Median UMI Counts per Cell
0	10769	39.38%	655	1113
1	2745	10.04%	458	875
2	2163	7.91%	622	1142
3	1471	5.38%	652	1296
4	1428	5.22%	631.5	1129
5	1025	3.75%	724	1464
6	884	3.23%	741.5	1290

7	702	2.57%	803.5	1843
8	694	2.54%	667	1023.5
9	666	2.44%	610	928
10	633	2.31%	743	1471
11	473	1.73%	686	1660
12	465	1.70%	696	1359
13	420	1.54%	625.5	1600.5
14	407	1.49%	599	1093
15	367	1.34%	610	1616
16	350	1.28%	588.5	886.5
17	343	1.25%	804	1475
18	285	1.04%	681	1154
19	273	0.99%	564	1072
20	259	0.95%	533	885
21	207	0.76%	774	1304
22	96	0.35%	865.5	1370.5
23	86	0.31%	589.5	1042.5
24	75	0.28%	676	1244
25	60	0.22%	784	1277

Table S5 Cell number and ratio of each sample in different cluster.

Cluster	Pa	Pc	Ga	Gc
Total	7910 (100%)	8839 (100%)	2792 (100%)	7805 (100%)
0	3189 (40.31%)	2763 (31.25%)	1267(45.38%)	3550 (44.48%)
1	691 (8.74%)	1287 (14.56%)	220 (7.88%)	547 (7.01%)
2	617 (7.80%)	539 (6.10%)	209 (7.49%)	798 (10.22%)
3	535 (6.76%)	386 (4.37%)	107 (3.83%)	443 (5.68%)
4	311 (3.93%)	635 (7.18%)	239 (8.56%)	243 (3.11%)
5	266 (3.36%)	733 (8.29%)	5 (0.18%)	21 (0.27%)
6	381 (4.82%)	195 (2.21%)	104 (3.72%)	204 (2.61%)
7	66 (0.83%)	355 (4.02%)	48 (1.72%)	233 (2.99%)
8	167 (2.11%)	284 (3.21%)	71 (2.54%)	172 (2.20%)
9	92 (1.16%)	306 (3.46%)	26 (0.93%)	242 (3.10%)
10	277 (3.50%)	162 (1.83%)	63 (2.26%)	131 (1.68%)
11	169 (2.14%)	179 (2.03%)	47 (1.68%)	78 (1.00%)
12	168 (2.12%)	145 (1.64%)	61 (2.18%)	91 (1.17%)
13	115 (1.45%)	131 (1.48%)	47 (1.68%)	127 (1.63%)
14	115 (1.45%)	116 (1.31%)	41 (1.47%)	135 (1.73%)

15	96 (1.21%)	81 (0.92%)	34 (1.22%)	156 (2.00%)
16	96 (1.21%)	90 (1.02%)	60 (2.15%)	104 (1.33%)
17	165 (2.09%)	43 (0.49%)	18 (0.64%)	117 (1.50%)
18	32 (0.40%)	101 (1.14%)	32 (1.15%)	120 (1.54%)
19	111 (1.40%)	70 (0.79%)	34 (1.22%)	58 (0.74%)
20	88 (1.11%)	82 (0.93%)	12 (0.43%)	77 (0.99%)
21	70 (0.88%)	80 (0.91%)	8 (0.29%)	49 (0.63%)
22	11 (0.14%)	24 (0.27%)	8 (0.29%)	53 (0.68%)
23	29 (0.37%)	27 (0.31%)	8 (0.29%)	22 (0.28%)
24	30 (0.38%)	15 (0.17%)	10 (0.36%)	20 (0.26%)
25	23 (0.29%)	10 (0.11%)	13 (0.47%)	14 (0.18%)

Table S6 Cell cluster annotations with the most representative marker genes.

Cluster	Cell type	Marker gene
9	Quinocyte	PKS1 (polyketide synthase)/AJAP22132, quinone oxidoreductase/AJAP00874
10	Skeletogenic cell	PKS2 (polyketide synthase 2)/AJAP05722
12, 18	Mesodermal cell	gataE (AJAP19786), GCM (AJAP20284)
5	Fibroblast	Astacin (AJAP21609), gated 1 (AJAP18379)
1	Melanocyte	Melanotransferrin (AJAP03036/ AJAP16542)
6	Neurons	Elav (AJAP05157)
2	Macrophage	CD163 (AJAP03614/ AJAP29225)
3	Tissue stem cell	Cyclin-L1 (AJAP00375), TNR (AJAP06757)
0，22	Epithelial cell	FGFR (AJAP00555)

Table S7 Differentially expressed genes (DEGs) in different comparison groups.

Cluster \ Group	Ga Vs Gc		Pa Vs Pc		Ga Vs Pa		Gc Vs Pc	
	Up	Down	Up	Down	Up	Down	Up	Down
bulk	422	414	353	408	398	1072	417	998
0	459	439	264	286	479	1289	549	1041
1	133	87	153	428	150	184	276	1004
2	169	128	78	122	123	130	265	730
3	30	20	40	84	74	46	93	233
4	83	32	128	135	126	107	119	277
5	0	0	111	163	0	0	4	10
6	10	9	43	63	62	26	38	49
7	8	5	16	11	5	5	39	97
8	8	5	24	54	51	20	44	135
9	0	0	33	103	0	2	66	216
10	3	5	20	16	15	32	16	40
11	1	2	26	25	9	12	29	35
12	4	3	14	25	17	10	14	28
13	5	1	10	10	2	4	13	25
14	2	1	4	9	12	5	11	31
15	2	2	4	11	5	10	18	46
16	12	3	7	16	15	11	10	24
17	0	0	3	2	1	3	6	20
18	0	0	3	3	0	0	17	24
19	1	1	13	15	9	3	4	9
20	1	0	5	7	1	0	8	16
21	0	0	3	1	0	0	6	15
22	0	0	0	0	0	0	0	1
23	0	0	0	1	0	0	1	0
24	0	0	0	0	0	0	0	0
25	0	0	0	0	0	0	0	0

Table S9 Acquisition parameters of melanin and quinone pigments.

Q1	Q3	RT（min）	Compound	DP	CE	CXP
317.2	149	4.66	Melanin	-126	-28	-10
172	172	3.96	Menadione	-110	-6	-11
173	145	4.12	Juglone 1	-100	-26	-10
173	116.8	4.12	Juglone 2	-100	-49	-10

172.7	145	4.20	2-Hydroxy-1,4-naphoquinone1	-87	-28	-8
172.7	100.9	4.20	2-Hydroxy-1,4-naphoquinone2	-60	-35	-6
188.9	161	3.85	5,8-Dihydroxy-1,4-naphthoquinone1	-126	-27	-13
188.9	145	3.85	5,8-Dihydroxy-1,4-naphthoquinone2	-107	-24	-11
187	97.3	4.15	2,7-Dimethyl-1,4-naphthoquinone	-85	-27	-6
