# Supplementary figures and images for: Single-Cell Transcriptome and Pigment Biochemistry Analysis Reveals the Potential for the High Nutritional and Medicinal Value of Purple Sea Cucumbers
Source: Int J Mol Sci. 2023 Jul 30;24(15):12213. doi: 10.3390/ijms241512213 (PMC10419132; doi:10.3390/ijms241512213)

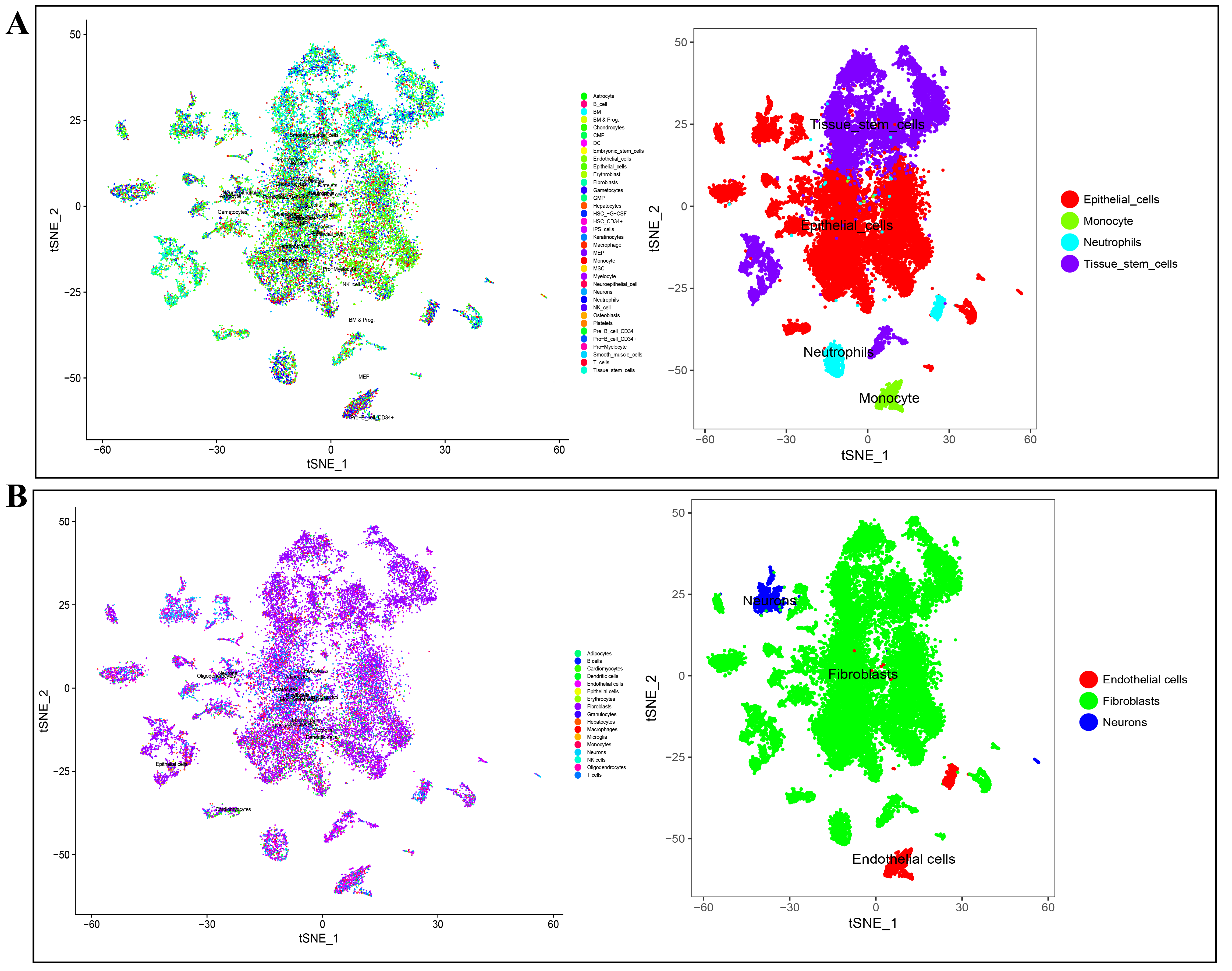

Supplement: Supplementary file 1 [file ijms-24-12213-s001.zip › Figure S1.tif]

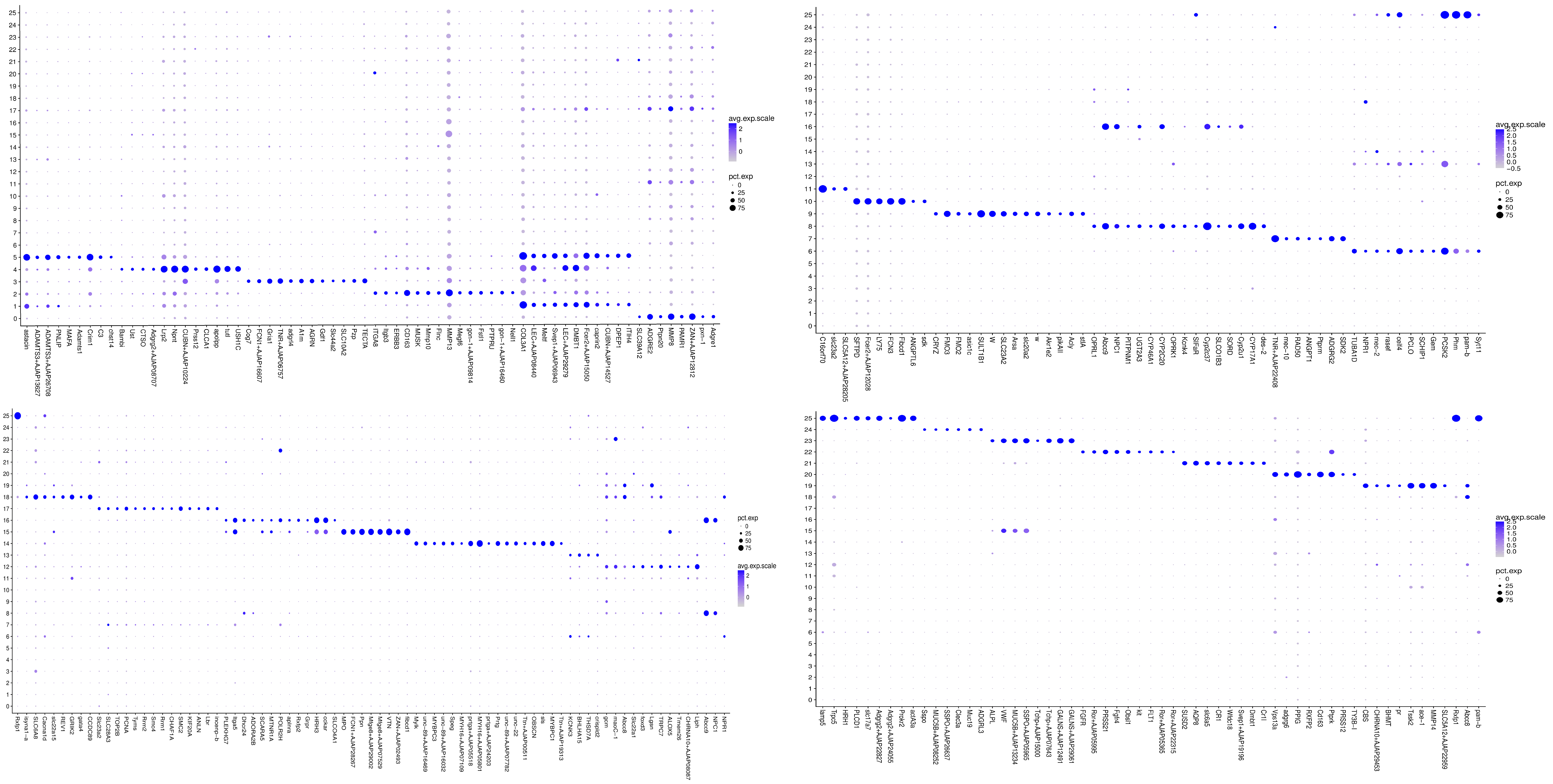

Supplement: Supplementary file 1 [file ijms-24-12213-s001.zip › Figure S2.tif]

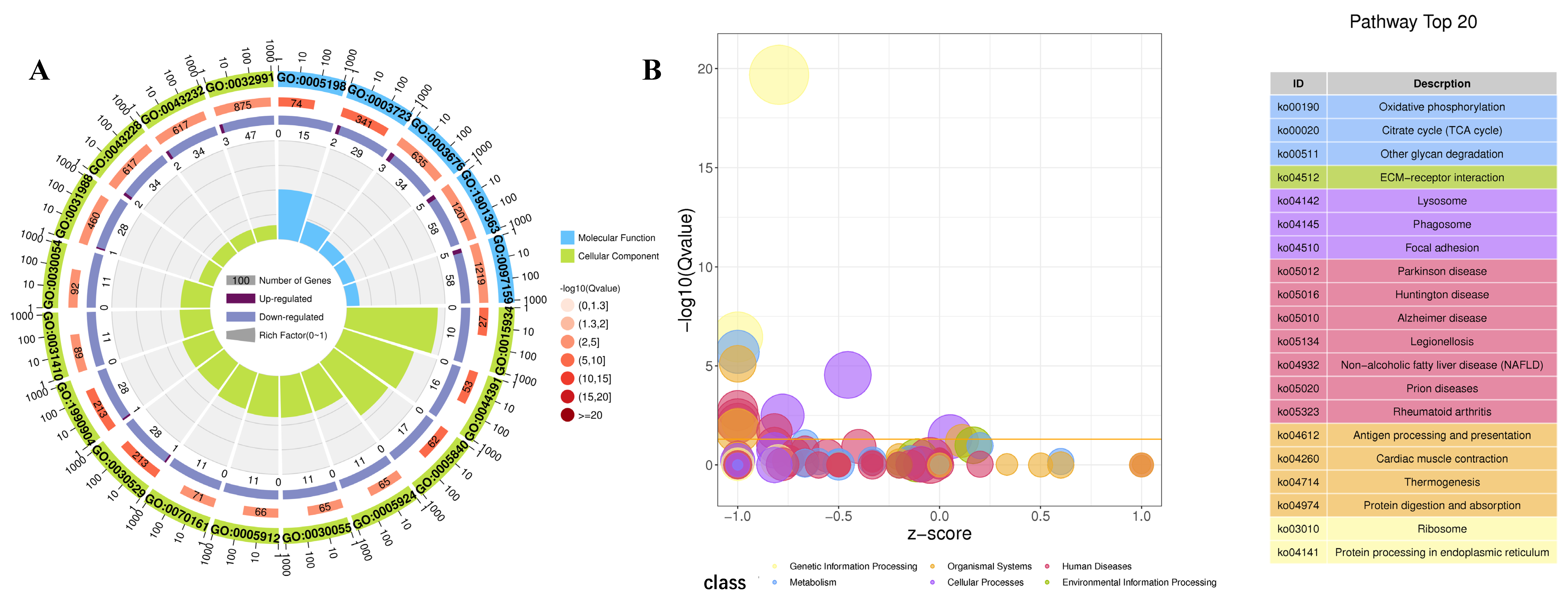

Supplement: Supplementary file 1 [file ijms-24-12213-s001.zip › Figure S3.tiff]

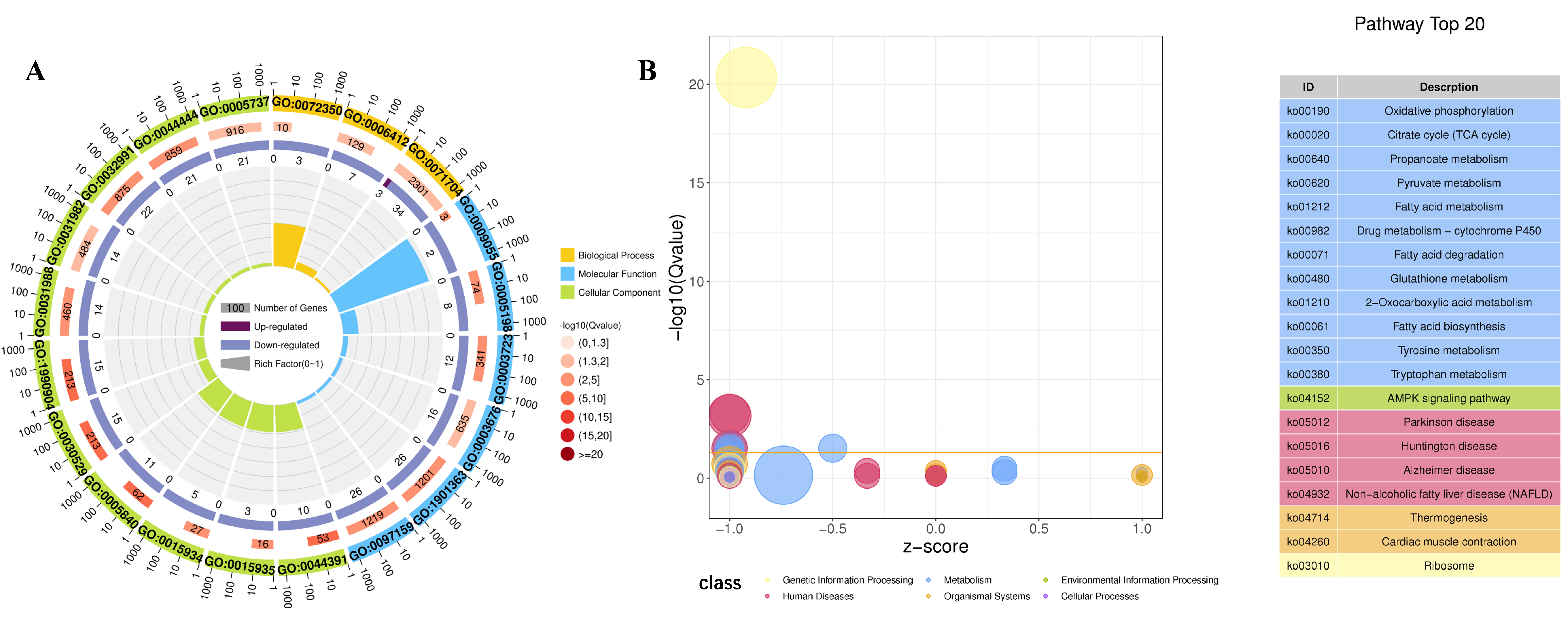

Supplement: Supplementary file 1 [file ijms-24-12213-s001.zip › Figure S4.tiff]

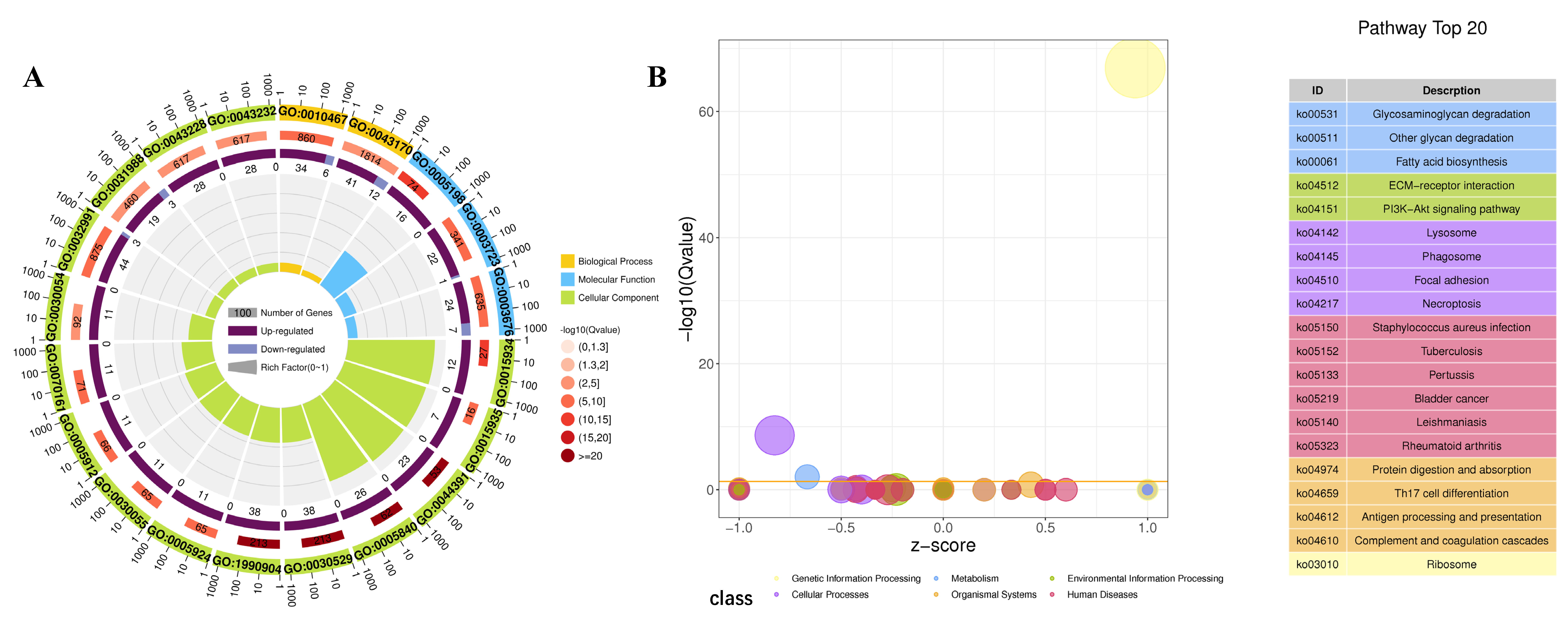

Supplement: Supplementary file 1 [file ijms-24-12213-s001.zip › Figure S5.tiff]

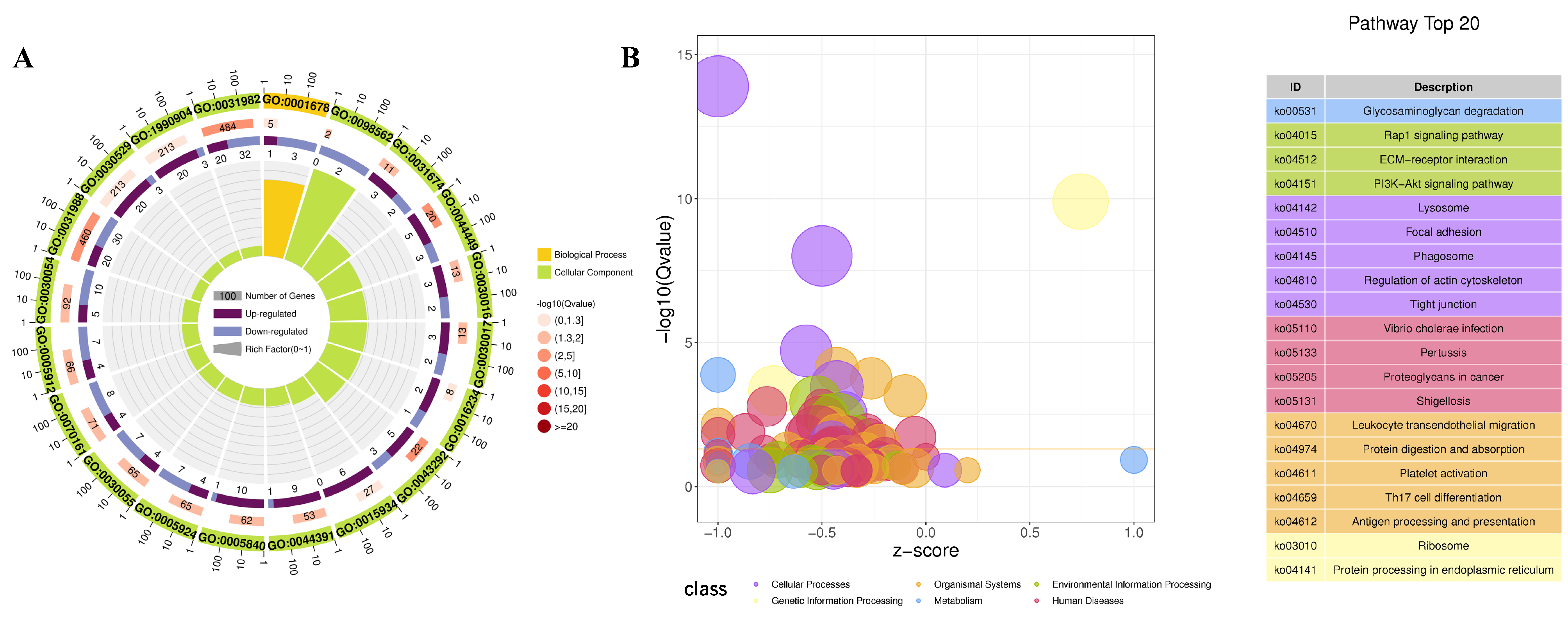

Supplement: Supplementary file 1 [file ijms-24-12213-s001.zip › Figure S6.tiff]

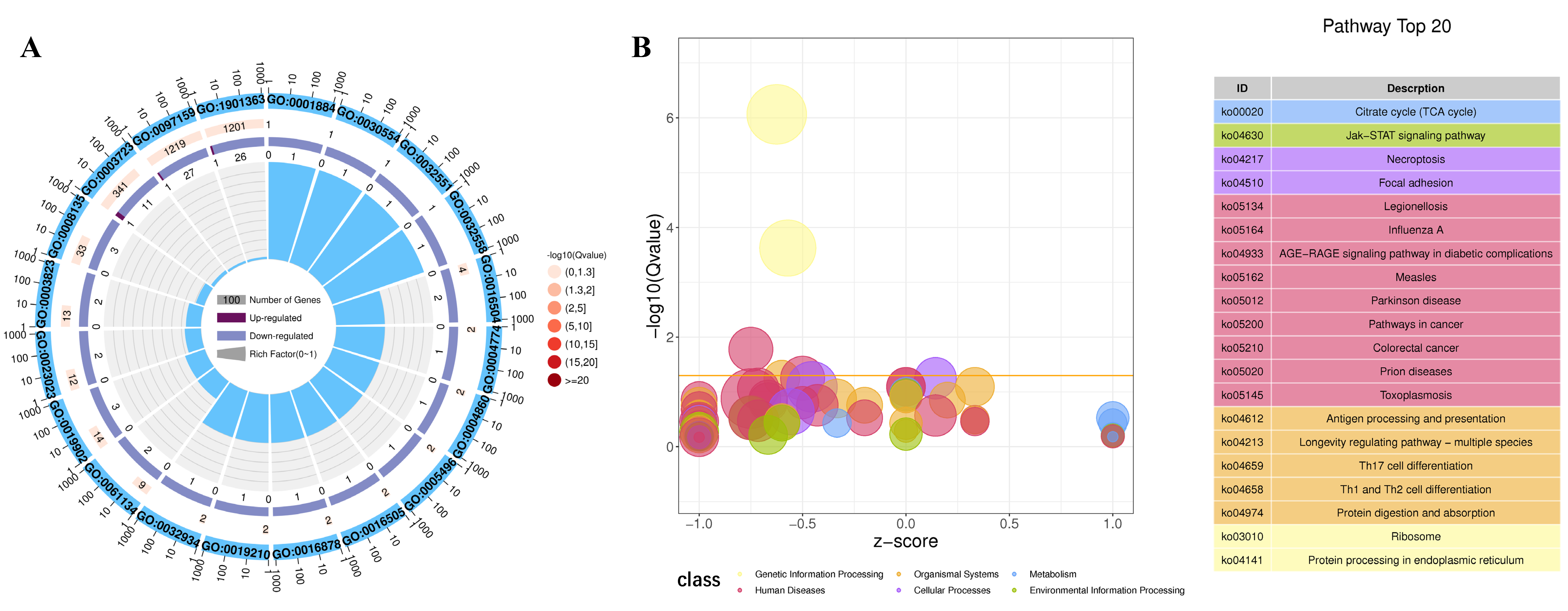

Supplement: Supplementary file 1 [file ijms-24-12213-s001.zip › Figure S7.tiff]
